# The Relationship Between Quadriceps Muscle and Tendon Morphology and Physical Performance in Patellofemoral Pain Syndrome

**DOI:** 10.3390/diagnostics16131984

**Published:** 2026-06-25

**Authors:** Mehmet Gök, Abdurrahim Tekin

**Affiliations:** 1 Department of Physical Medicine and Rehabilitation, Bingöl State Hospital, Bingöl 12000, Türkiye; mgok19866@gmail.com; 2 Department of Neurosurgery, Kanuni Sultan Süleyman Training and Research Hospital, University of Health Sciences, Istanbul 34303, Türkiye

**Keywords:** patellofemoral pain syndrome, ultrasonography, quadriceps muscle, tendons, physical functional performance, muscle, skeletal

## Abstract

**Objective:** Patellofemoral pain syndrome (PFPS) is one of the most common causes of anterior knee pain and is associated with biomechanical, muscular, and functional impairments affecting the extensor mechanism of the knee. Quadriceps muscle dysfunction, altered tendon morphology, and impaired lower extremity biomechanics have been suggested to contribute to patellofemoral joint instability and pain development. The aim of this study was to evaluate the muscle and tendon thicknesses of the extensor mechanism using ultrasonography in individuals with PFPS and to investigate the relationship of these measurements with knee pain, knee function, and physical performance, with particular emphasis on the combined assessment of muscle morphology, tendon morphology, and functional performance parameters. **Methods:** This cross-sectional study was conducted between 5 November 2019 and 15 December 2019, including 80 individuals aged 18–45 years who presented with anterior knee pain and were clinically diagnosed with patellofemoral pain syndrome (PFPS). Demographic characteristics of the participants were collected. Pain severity was assessed using the Visual Analog Scale (VAS), and functional status was evaluated with the Western Ontario and McMaster Universities Osteoarthritis Index (WOMAC). Physical performance was assessed using the 6 m walk test and the five-repetition sit-to-stand test. Ultrasonographic examination was used to measure rectus femoris muscle thickness, vastus intermedius muscle thickness, quadriceps tendon thickness, and patellar tendon thickness using a high-frequency linear probe in a standardized supine position with the knee relaxed and the lower extremity muscles at rest. **Results:** The mean age of the participants was 32.11 ± 7.08 years, and the mean body mass index (BMI) was 25.05 ± 4.11 kg/m^2^. Of the participants, 42 (52.5%) were male and 38 (47.5%) were female; 46 (57.5%) were smokers and 34 (42.5%) were non-smokers. Ultrasonographic measurements showed that rectus femoris muscle thickness was 1.98 ± 0.45 cm, vastus intermedius muscle thickness was 1.75 ± 0.53 cm, quadriceps tendon thickness was 0.54 ± 0.12 cm, and patellar tendon thickness was 0.35 ± 0.08 cm. Rectus femoris, vastus intermedius, and quadriceps tendon thicknesses were significantly higher in males compared to females (*p* = 0.001). Individuals with BMI > 25 had greater rectus femoris (*p* = 0.023) and vastus intermedius (*p* = 0.001) muscle thicknesses. A negative correlation was found between rectus femoris muscle thickness and WOMAC total (r = −0.227, *p* = 0.042) and WOMAC pain scores (r = −0.233, *p* = 0.028). Additionally, a significant relationship was observed between quadriceps tendon thickness and the five-repetition sit-to-stand test (r = −0.247, *p* = 0.044). **Conclusions:** In patients with PFPS, quadriceps muscle and tendon thicknesses were found to be associated with certain demographic and clinical parameters. Ultrasonographic evaluation of muscle and tendon structures may be a useful, non-invasive, dynamic, and radiation-free method for better understanding the clinical characteristics of PFPS and its relationship with physical performance. Ultrasonographic assessment may also provide complementary information for rehabilitation planning and functional evaluation in individuals with PFPS, although these findings should be interpreted cautiously because of the cross-sectional design and weak correlations observed.

## 1. Introduction

Patellofemoral pain syndrome (PFPS) is one of the most common causes of anterior knee pain and is characterized by diffuse peri- or retro-patellar pain that increases during activities such as stair climbing, squatting, prolonged sitting, running, and jumping [1,2,3,4,5]. PFPS is highly prevalent in both physically active individuals and the general population, with a particularly increased incidence among young adults, athletes, and females [2,3,4,5,6]. The prevalence of PFPS has been reported to range between 7% and 28% in the general population, while higher rates have been demonstrated in military recruits and athletic populations [3,4,5,6].

Although PFPS has been extensively investigated, its etiology remains multifactorial and incompletely understood [1,2,3,4,5,6]. Several intrinsic and extrinsic factors have been associated with the development of PFPS, including abnormal lower extremity biomechanics, altered patellar tracking, excessive dynamic valgus, increased Q-angle, femoral internal rotation, foot pronation, muscle weakness, soft tissue tightness, and neuromuscular imbalance [2,3,4,5,6,7]. Therefore, PFPS should not be considered only as a pain condition, but also as a disorder that may involve structural, muscular, biomechanical, and functional components of the knee extensor mechanism. Previous biomechanical studies have suggested that altered force distribution across the patellofemoral joint may increase joint stress and contribute to pain generation and functional impairment [2,4,5,7].

Among the muscular factors associated with PFPS, quadriceps dysfunction has received particular attention. The quadriceps femoris muscle group plays a crucial role in maintaining patellofemoral joint stability and controlling patellar tracking during dynamic activities [4,7,8,9,10]. Altered quadriceps activation patterns, delayed vastus medialis obliquus activation, decreased muscle strength, reduced muscle thickness, impaired muscle quality, altered echogenicity, proximal and distal muscle morphology changes, and knee crepitus-related muscle alterations have all been associated with PFPS [7,8,9,10,11,12,13,14,15,16,17]. Recent evidence has also suggested that quadriceps isometric strength may be related to knee proprioception in individuals with PFPS, further supporting the functional importance of quadriceps assessment in this population [18]. However, muscle morphology and tendon morphology are often evaluated separately, and the functional relevance of these ultrasonographic measurements remains insufficiently clarified in patients with PFPS. Furthermore, abnormalities in tendon morphology, quadriceps fat pad morphology, and patellofemoral structural alterations may influence extensor mechanism biomechanics and contribute to functional limitations and pain severity [8,10,11,12,19].

Recent studies have demonstrated that ultrasonographic assessment of quadriceps muscle morphology may provide clinically valuable information in patients with PFPS [8,9,10,11,12,13,14,15]. Ultrasonography is a non-invasive, radiation-free, dynamic, and cost-effective imaging modality that allows real-time evaluation of muscle and tendon structures. Compared with magnetic resonance imaging (MRI), ultrasonography offers several practical advantages, including accessibility, lower cost, portability, and dynamic examination capability [10,13,14,20]. Previous studies have reported that ultrasonographic measurements of quadriceps muscle thickness correlate with muscle strength, muscle function, and clinical symptoms in PFPS [8,10,11,15,21]. Nevertheless, available studies have mostly focused on isolated sonographic parameters, comparisons between PFPS and healthy controls, or specific muscle components. Fewer studies have examined whether ultrasonographic measurements of both muscle and tendon thickness are associated with patient-reported pain, functional status, and simple physical performance tests within the same PFPS cohort. In addition, sonographic evaluation has been used to assess tendon morphology, muscle echogenicity, fiber angle, muscle activation patterns, and structural adaptations associated with rehabilitation interventions [9,12,13,14,15].

Physical performance impairment is another important component of PFPS. Patients frequently experience difficulty during activities requiring repetitive knee flexion and extension, which may negatively affect quality of life and physical activity participation [1,3,4,5]. Functional tests such as walking assessments and sit-to-stand tests are commonly used to evaluate lower extremity performance in patients with PFPS, while updated physical examination approaches may improve clinical assessment and diagnostic accuracy [19]. Although WOMAC is more commonly used in osteoarthritis populations, its pain and function subscales may provide complementary information regarding knee-related symptoms and functional limitations when interpreted cautiously in PFPS. In addition, clinical scales including the Visual Analog Scale (VAS) and Western Ontario and McMaster Universities Osteoarthritis Index (WOMAC) provide valuable information regarding pain severity and functional limitations [1,3,4].

Despite increasing interest in ultrasonographic evaluation of quadriceps morphology in PFPS, data regarding the relationship between muscle and tendon thicknesses and physical performance parameters remain limited. Unlike previous studies that primarily examined isolated quadriceps muscle characteristics, tendon thickness, or case-control differences, the present study evaluates both muscle and tendon thicknesses together and relates these measurements to pain, self-reported function, and physical performance in the same PFPS cohort [8,9,10,11,12,13,14,15]. Therefore, the aim of this study was to evaluate the muscle and tendon thicknesses of the extensor mechanism using ultrasonography in individuals with PFPS and to investigate the relationship of these measurements with knee pain, knee function, and physical performance.

## 2. Materials and Methods

### 2.1. Study Design and Participants

This cross-sectional study was conducted between 5 November 2019 and 15 December 2019 and included individuals aged 18–45 years who presented with anterior knee pain and were clinically diagnosed with PFPS based on clinical history and physical examination findings. The study was reported in accordance with the Strengthening the Reporting of Observational Studies in Epidemiology (STROBE) recommendations for observational cross-sectional studies. The study was carried out at the outpatient clinic of the Department of Physical Medicine and Rehabilitation, Hacettepe University Faculty of Medicine. A total of 80 patients without any systemic, inflammatory, rheumatologic, or neurological disease were evaluated.

PFPS was diagnosed clinically as a diagnosis of exclusion. Symptom characteristics, including pain location, aggravating activities, and symptom duration, were obtained from the clinical history. Patients were eligible if they had anterior, retropatellar, or peripatellar knee pain that was aggravated by patellofemoral joint-loading activities, such as stair climbing, squatting, prolonged sitting with the knee flexed, running, jumping, kneeling, or similar activities involving repetitive knee flexion and extension. Clinical diagnosis was supported by physical examination findings, including patellofemoral pain provocation during the patellar grinding test and/or Clarke test, assessment of patellar tilt, Q-angle measurement, and evaluation of soft tissue tightness involving the iliotibial band, hamstrings, and quadriceps muscles.

Participants with previous knee surgery, a history of significant knee trauma, inflammatory arthritis, neurological disorders affecting lower extremity function, or other causes of anterior knee pain were excluded from the study. Other potential causes of anterior knee pain were considered during clinical evaluation and excluded when suspected. These included meniscal injury, ligamentous instability, patellar dislocation or subluxation, patellar or quadriceps tendinopathy, osteoarthritis, intra-articular knee pathology, referred pain from the hip or lumbar spine, systemic inflammatory disease, rheumatologic disease, and neurological or musculoskeletal disorders that could affect walking or sit-to-stand performance. Demographic characteristics of the participants, including age, sex, height, body weight, body mass index (BMI), and smoking status, were recorded.

### 2.2. Clinical and Physical Evaluation

Clinical tests related to the patellofemoral joint were evaluated during physical examination. The patellar tilt test was used to assess the lateral retinacular tightness of the patella. The presence of patellofemoral joint-related pain was investigated using the patellar grinding test and Clarke test. In addition, Q-angle measurement was performed to evaluate lower extremity biomechanics, and the presence of tightness in the iliotibial band, hamstring, and quadriceps muscles was assessed.

Pain severity was evaluated using the Visual Analog Scale (VAS). The VAS was used as a 0–10 scale, with higher scores indicating greater pain intensity. Functional status was assessed using the Western Ontario and McMaster Universities Osteoarthritis Index (WOMAC). Although WOMAC is more commonly used in osteoarthritis populations, it was used in the present study to obtain standardized information on knee-related pain, stiffness, and functional limitation. Therefore, WOMAC results were interpreted as complementary patient-reported functional data rather than as disease-specific PFPS outcome measures. WOMAC total score and WOMAC pain score were included in the analyses.

Physical performance was evaluated using the 6 m walk test and the five-repetition sit-to-stand test. During the walking test, participants were instructed to complete the designated distance at their normal walking speed, and the completion time was recorded. In the five-repetition sit-to-stand test, participants were asked to stand up and sit down five times consecutively from a standard-height chair as quickly as possible without upper extremity support, and the completion time was recorded in seconds. Both tests were selected because they are simple, reproducible, and clinically applicable performance-based assessments reflecting walking ability and lower extremity functional capacity.

### 2.3. Ultrasonographic Evaluation

Ultrasonographic examination was performed to evaluate the muscle and tendon morphology of the extensor mechanism. All ultrasonographic assessments were conducted by an experienced radiologist specialized in musculoskeletal ultrasonography using a high-frequency linear probe with an ultrasound device (Philips Affiniti 70G, Philips Healthcare, Bothell, WA, USA). Participants were evaluated in the supine position with the knee in a relaxed position and the lower extremity muscles at rest.

Rectus femoris muscle thickness, vastus intermedius muscle thickness, quadriceps tendon thickness, and patellar tendon thickness were measured using standard anatomical reference points. Muscle measurements were obtained in the longitudinal plane, and care was taken to avoid excessive probe compression during evaluation. Tendon morphology and thickness were also assessed sonographically.

All measurements were performed on the symptomatic knee. In patients with bilateral symptoms, the more symptomatic side was evaluated. Participants were positioned supine with the knee relaxed and extended, and the quadriceps muscle was kept at rest during image acquisition. A sufficient amount of gel was used, and minimal probe pressure was applied to avoid compression-related measurement error.

Rectus femoris and vastus intermedius muscle thicknesses were assessed in the anterior thigh using a longitudinal probe orientation over the quadriceps muscle. Muscle thickness was measured as the vertical distance between the superficial and deep fascial borders of the relevant muscle. Quadriceps tendon thickness was measured in the longitudinal plane approximately 1 cm proximal to the superior pole/apex of the patella, and patellar tendon thickness was measured in the longitudinal plane approximately 1 cm distal to the inferior pole/apex of the patella.

For each ultrasonographic variable, measurements were repeated three times, and the mean value was used for statistical analysis. Images were obtained under standardized conditions by the same experienced examiner to reduce operator-related variability. Formal intra-observer and inter-observer reliability analyses were not performed in the original dataset; therefore, the lack of reliability testing was considered a methodological limitation.

### 2.4. Statistical Analysis

Statistical analyses were performed using SPSS software version 23.0 (IBM Corp., Armonk, NY, USA). Categorical variables were expressed as numbers and percentages, while continuous variables were presented as mean ± standard deviation and minimum–maximum values. The normality of numerical data was assessed using the Kolmogorov–Smirnov and Shapiro–Wilk tests.

For comparisons between two groups, the Student’s t-test was used for variables with normal distribution, and the Mann–Whitney U test was used for variables without normal distribution. Relationships between variables were evaluated using Spearman correlation analysis. Multiple linear regression analysis using the backward selection method was performed to evaluate the effects of independent variables on clinical and physical performance outcomes. Statistical significance was accepted as *p* < 0.05.

Categorical variables were compared using the chi-square test or Fisher’s exact test when appropriate. Because several clinical and ultrasonographic variables were not normally distributed, non-parametric analyses were preferred for correlation analyses. Spearman correlation coefficients were interpreted according to their magnitude, and weak correlations were not considered sufficient to indicate clinical usefulness or predictive value.

Sex, BMI category, and smoking status were evaluated as potential factors associated with ultrasonographic measurements using subgroup comparisons. Regression analyses were performed to explore whether selected ultrasonographic and clinical variables were associated with pain, function, and physical performance outcomes. Because this was an observational cross-sectional study with a limited sample size and no healthy control group, the analyses were considered exploratory. No formal a priori sample size calculation was available in the original dataset; therefore, the possibility of limited statistical power was acknowledged.

### 2.5. Ethical Approval

This study was conducted in accordance with the principles of the Declaration of Helsinki. The study protocol was approved by the Hacettepe University Non-Interventional Clinical Research Ethics Committee (approval number: GO 19/993; approval date: 5 November 2019). All participants were informed in detail about the study protocol, and written informed consent was obtained prior to participation. Participant data were evaluated anonymously in accordance with confidentiality principles.

## 3. Results

### 3.1. Demographic and Clinical Characteristics

A total of 80 patients with PFPS were included in the study. The study population consisted of 42 males (52.5%) and 38 females (47.5%). Forty-six patients (57.5%) were smokers, whereas 34 patients (42.5%) were non-smokers. The mean age of the participants was 32.11 ± 7.08 years (20–45 years), and the mean BMI was 25.05 ± 4.11 kg/m^2^ (17–36 kg/m^2^). The mean VAS score was 4.60 ± 2.23, the mean WOMAC pain score was 6.40 ± 3.86, and the mean WOMAC total score was 25.14 ± 16.73. The mean 6 m walk test time was 5.12 ± 1.03 s, and the mean five-repetition sit-to-stand test time was 12.46 ± 2.41 s (Table 1).

### 3.2. Ultrasonographic Findings

In the ultrasonographic evaluation, the mean rectus femoris muscle thickness was 1.98 ± 0.45 cm (1.01–3.01 cm), while the mean vastus intermedius muscle thickness was 1.75 ± 0.53 cm (0.80–3.11 cm). The mean quadriceps tendon thickness was 0.54 ± 0.12 cm (0.31–0.92 cm), and the mean patellar tendon thickness was 0.35 ± 0.08 cm (0.23–0.80 cm) (Table 2).

**Figure 1:**
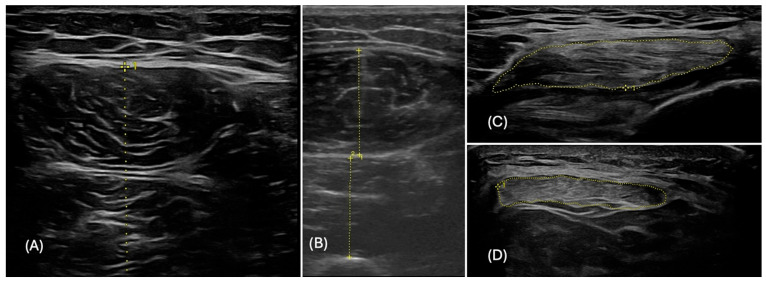
Representative ultrasonographic images obtained during ultrasonographic evaluation. (**A**,**B**) Measurement of rectus femoris and vastus intermedius muscle thicknesses. (**C**) Ultrasonographic appearance and contouring of the quadriceps tendon. (**D**) Ultrasonographic appearance and contouring of the patellar tendon. The yellow dotted lines indicate the muscle thickness measurements, and the yellow contours indicate the tendon boundaries.

Representative ultrasonographic images obtained during ultrasonographic evaluation (Figure 1).

### 3.3. Comparison According to Sex, BMI, and Smoking Status

In analyses based on sex, rectus femoris muscle thickness was significantly higher in males (2.25 ± 0.37 cm) compared to females (1.69 ± 0.35 cm) (*p* = 0.001). Similarly, vastus intermedius muscle thickness was 1.94 ± 0.55 cm in males and 1.55 ± 0.44 cm in females, and this difference was statistically significant (*p* = 0.001). Quadriceps tendon thickness was also significantly higher in males (0.60 ± 0.13 cm) than in females (0.49 ± 0.10 cm) (*p* = 0.001). Patellar tendon thickness was also significantly higher in males (0.40 ± 0.09 cm) than in females (0.31 ± 0.05 cm) (*p* = 0.001). However, no significant sex-based differences were observed in the 6 m walk test, five-repetition sit-to-stand test, VAS score, WOMAC total score, WOMAC pain score, WOMAC stiffness score, or WOMAC function score (Table 3).

When the participants were evaluated according to BMI, rectus femoris muscle thickness was significantly higher in individuals with BMI > 25 compared to those with BMI ≤ 25 (2.10 ± 0.45 cm vs. 1.88 ± 0.44 cm, respectively; *p* = 0.023). Likewise, vastus intermedius muscle thickness was significantly greater in the BMI >25 group (2.01 ± 0.54 cm) than in the BMI ≤ 25 group (1.53 ± 0.42 cm) (*p* = 0.001). No statistically significant difference was found between BMI groups regarding quadriceps tendon thickness (*p* = 0.224). Patellar tendon thickness was significantly higher in patients with BMI > 25 kg/m^2^ than in those with BMI ≤ 25 kg/m^2^ (*p* = 0.028). Quadriceps tendon thickness, physical performance tests, VAS score, and WOMAC scores did not differ significantly according to BMI category (Table 4).

Rectus femoris muscle thickness was significantly higher in smokers compared to non-smokers (2.10 ± 0.41 cm vs. 1.90 ± 0.48 cm, respectively; *p* = 0.047). Similarly, vastus intermedius muscle thickness was greater in smokers than in non-smokers (1.91 ± 0.57 cm vs. 1.64 ± 0.48 cm, respectively; *p* = 0.041). However, quadriceps tendon thickness did not significantly differ according to smoking status (*p* = 0.148). No significant smoking-related differences were observed in patellar tendon thickness, physical performance tests, VAS score, or WOMAC scores (Table 5).

### 3.4. Correlation Analyses

Correlation analyses demonstrated a significant negative correlation between rectus femoris muscle thickness and WOMAC total score (r = −0.227, *p* = 0.042). In addition, rectus femoris muscle thickness was negatively correlated with WOMAC pain score (r = −0.233, *p* = 0.028). Rectus femoris thickness also showed a borderline negative correlation with VAS score (r = −0.218, *p* = 0.052). A significant relationship was also found between quadriceps tendon thickness and five-repetition sit-to-stand test performance (r = −0.247, *p* = 0.044). No significant correlations were observed between patellar tendon thickness and the clinical or physical performance outcomes (Table 6).

### 3.5. Regression Analyses

Regression analyses were performed to explore the associations between selected ultrasonographic and clinical variables and functional outcomes. In the regression model for the 6 m walk test, the five-repetition sit-to-stand test was significantly associated with 6 m walk test time (β = −0.302, *p* = 0.007, R^2^ = 0.091). In the model for the five-repetition sit-to-stand test, 6 m walk test time (β = −0.286, *p* = 0.008) and quadriceps tendon thickness (β = −0.215, *p* = 0.044) were significant predictors, with an overall R^2^ value of 0.174.

Rectus femoris thickness showed a borderline association with VAS score (β = −0.218, *p* = 0.052, R^2^ = 0.047). Rectus femoris thickness was significantly associated with WOMAC pain score (β = −0.252, *p* = 0.028, R^2^ = 0.064) and WOMAC total score (β = −0.258, *p* = 0.042, R^2^ = 0.066). However, the explanatory power of these models was low, indicating that these findings should be interpreted as weak exploratory associations rather than strong predictive relationships (Table 7).

## 4. Discussion

The main findings were that rectus femoris thickness showed weak negative associations with WOMAC pain and total scores, quadriceps tendon thickness showed a weak negative association with five-repetition sit-to-stand test time, and sex, BMI, and smoking status were associated with selected ultrasonographic measurements. Overall, these findings indicate weak exploratory associations rather than strong predictive relationships. PFPS is considered a multifactorial disorder involving biomechanical, muscular, structural, and functional alterations affecting the patellofemoral joint [1,2,3,4,5]. Previous studies have shown that abnormal patellar tracking, altered lower extremity biomechanics, excessive dynamic valgus, hip muscle weakness, and quadriceps dysfunction may contribute to increased patellofemoral joint stress and symptom development [2,3,4,5,6,19]. Among these factors, quadriceps morphology and neuromuscular control appear to play important roles in maintaining patellofemoral stability during functional activities such as walking, stair climbing, and squatting [2,7,8,9,10]. Previous ultrasonographic and biomechanical studies have also demonstrated that reduced muscle strength, altered echogenicity, impaired muscle quality, and changes in muscle morphology may contribute to functional impairment in individuals with PFPS [7,8,9,10,15,16,17]. Recent evidence has also suggested that quadriceps strength may be associated with knee proprioception in individuals with PFPS, further supporting the functional relevance of quadriceps assessment in this population [18]. Unlike previous studies that primarily focused on isolated muscle parameters, muscle strength, echogenicity, or comparisons with healthy controls, the present study evaluated both muscle and tendon thicknesses and examined their associations with patient-reported symptoms and simple physical performance tests within the same PFPS cohort [8,9,10,15,16,17].

In the present study, rectus femoris and vastus intermedius muscle thicknesses were significantly higher in males compared to females. This finding is consistent with previous reports demonstrating sex-related differences in quadriceps muscle morphology and strength [2,4,6,7]. Females are known to have a higher prevalence of PFPS, and anatomical and biomechanical differences such as increased pelvic width, greater valgus tendency, and altered neuromuscular control may partially explain this increased susceptibility [3,6]. In addition, increased BMI was associated with greater rectus femoris and vastus intermedius muscle thicknesses. Increased body weight may lead to adaptive muscular changes because of greater mechanical loading across the lower extremities. Similar associations between body composition and quadriceps morphology have also been reported in previous PFPS studies [7,8,16]. In the present study, smoking status was also associated with rectus femoris and vastus intermedius thicknesses. However, because these subgroup comparisons were exploratory and not adjusted for all potential confounders, sex-, BMI-, and smoking-related differences should be interpreted cautiously. These factors may influence muscle and tendon morphology independently of PFPS.

Rectus femoris muscle thickness demonstrated a negative correlation with WOMAC total and WOMAC pain scores. This finding suggests that lower rectus femoris thickness may be weakly related to greater self-reported pain and functional limitation in individuals with PFPS. Similar observations have been reported in previous studies demonstrating relationships between quadriceps morphology, muscle strength, and functional impairment in PFPS [8,10,11,15]. Nevertheless, the magnitude of these correlations was small, and the regression models explained only a limited proportion of the variance in clinical outcomes. Therefore, rectus femoris thickness should not be interpreted as an independent predictor of pain or functional status, but rather as one component of the multifactorial clinical presentation of PFPS [1,2,3,4,5,13].

Quadriceps tendon thickness showed a weak negative association with five-repetition sit-to-stand test time. Because the sit-to-stand task requires coordinated activation of the quadriceps muscle group and the knee extensor mechanism, tendon morphology may have some relationship with functional task performance [6,7,13]. However, this association was weak, and the explanatory power of the regression model was low. Accordingly, quadriceps tendon thickness should not be considered an independent marker of functional capacity, but rather a complementary ultrasonographic parameter that may be interpreted together with clinical examination findings, patient-reported outcomes, and physical performance tests.

Recent studies have demonstrated the increasing role of ultrasonography in the evaluation of PFPS-related muscle and tendon abnormalities [8,9,10,11,12,13,14,15,20]. Ultrasonography is considered a potentially useful, non-invasive, radiation-free, dynamic, and cost-effective imaging modality for evaluating the extensor mechanism. Compared with MRI, ultrasonography offers several practical advantages, including lower cost, portability, accessibility, and dynamic examination capability [14,20]. Previous studies have also shown that ultrasonographic measurements may correlate with muscle strength, tendon morphology, echogenicity, and functional outcomes in patients with PFPS [8,9,10,11,12,13,14,15]. Therefore, ultrasonographic evaluation may provide complementary information during the clinical assessment of PFPS; however, the findings of the present study do not support the use of ultrasonographic thickness measurements as standalone diagnostic or prognostic markers.

An important point in interpreting the present findings is the absence of a healthy control group. Previous studies comparing patients with PFPS and healthy individuals have reported differences in quadriceps morphology, muscle function, or ultrasonographic parameters [8,10,14,15,16,17]. Because the present study included only individuals with PFPS, the results do not allow direct comparisons between patients with PFPS and asymptomatic individuals. Therefore, the present findings cannot establish whether the observed muscle or tendon thickness values are disease-specific morphological alterations. Instead, the results describe associations between ultrasonographic morphology and clinical–functional outcomes within a PFPS cohort.

The use of WOMAC in the present study also requires careful interpretation. WOMAC is traditionally used in patients with knee osteoarthritis; however, its pain and physical function domains can provide complementary information regarding knee-related symptoms and functional limitations. In this study, WOMAC was not used as a disease-specific PFPS scale, but rather as a standardized patient-reported measure of pain-related functional difficulty. Nevertheless, PFPS-specific outcome measures, such as the Kujala Anterior Knee Pain Scale, may be more appropriate for evaluating anterior knee pain and patellofemoral function in young and active populations [3,4,12,13]. Therefore, the use of WOMAC instead of a PFPS-specific scale should be considered a limitation of the study.

The present study has several strengths. First, it evaluated both quadriceps muscle thickness and tendon thickness in the same PFPS cohort, allowing a broader assessment of the knee extensor mechanism than studies focusing on isolated muscle or tendon parameters. Second, ultrasonographic measurements were examined together with pain severity, patient-reported functional status, and simple physical performance tests. Third, potential differences according to sex, BMI, and smoking status were explored, which may help contextualize the interpretation of muscle and tendon thickness measurements. These features may increase the descriptive value of the study despite its methodological limitations.

This study has several limitations. The cross-sectional design limits the ability to establish causal relationships between ultrasonographic findings and clinical parameters. In addition, the study was conducted in a single center with a relatively limited sample size, and a healthy control group was not included. Although sex, BMI, and smoking status were evaluated, other potential confounding factors, such as physical activity level, sports participation, occupational loading, lower extremity alignment, muscle strength, and rehabilitation history, could not be fully controlled. No formal a priori sample size calculation was available in the original dataset, and the relatively limited sample size may have reduced the statistical power to detect small associations. Formal intra-observer and inter-observer reliability analyses were not performed for ultrasonographic measurements, which limits the ability to determine measurement reproducibility. Finally, the observed correlations and regression models showed low explanatory power; therefore, the findings should be interpreted as exploratory and hypothesis-generating rather than definitive evidence of clinically predictive relationships. Future prospective controlled studies using PFPS-specific outcome measures, standardized reliability testing, direct strength assessment, and comprehensive biomechanical evaluation are needed to clarify the clinical relevance of quadriceps muscle and tendon morphology in PFPS.

## 5. Conclusions

In this study, quadriceps muscle and tendon thicknesses were evaluated using ultrasonography in individuals with PFPS, and these measurements were shown to be associated with certain demographic and clinical parameters. Quadriceps muscle thicknesses were found to be higher in males, some muscle thicknesses increased with higher BMI, and muscle thicknesses were associated with pain and functional performance parameters. These findings suggest that ultrasonographic evaluation of the muscle and tendon structures of the extensor mechanism in PFPS may contribute to a better understanding of the clinical characteristics of the condition. Ultrasonography may serve as a practical and complementary imaging method during clinical and functional assessment in individuals with PFPS. However, because the observed associations were weak and the study had a cross-sectional design without a healthy control group or formal reliability analysis, ultrasonographic thickness measurements should not be interpreted as independent diagnostic, prognostic, or treatment-guiding markers. Future prospective controlled studies using PFPS-specific outcome measures, standardized ultrasonographic protocols, reliability testing, and direct strength assessment are needed to clarify the clinical relevance of quadriceps muscle and tendon morphology in PFPS.

## Figures and Tables

**Table 1:** Baseline demographic, clinical, and physical performance characteristics of the participants.

Variable	Mean ± SD	Min–Max
Age (years)	32.11 ± 7.08	20–45
BMI (kg/m^2^)	25.05 ± 4.11	17–36
Number of participants	80	–
Sex, male/female	42 (52.5%)/38 (47.5%)	–
Smoking, yes/no	46 (57.5%)/34 (42.5%)	–
VAS score	4.60 ± 2.23	–
WOMAC pain score	6.40 ± 3.86	–
WOMAC total score	25.14 ± 16.73	–
6 m walk test, s	5.12 ± 1.03	–
Five-repetition sit-to-stand test, s	12.46 ± 2.41	–

SD: standard deviation; BMI: body mass index; VAS: Visual Analog Scale; WOMAC: Western Ontario and McMaster Universities Osteoarthritis Index.

**Table 2:** Ultrasonographic measurement results.

Measurement	Mean ± SD	Min–Max
Rectus femoris muscle thickness (cm)	1.98 ± 0.45	1.01–3.01
Vastus intermedius muscle thickness (cm)	1.75 ± 0.53	0.80–3.11
Quadriceps tendon thickness (cm)	0.54 ± 0.12	0.31–0.92
Patellar tendon thickness (cm)	0.35 ± 0.08	0.23–0.80

SD: standard deviation.

**Table 3:** Comparison of ultrasonographic, clinical, and physical performance variables according to sex.

Variable	Female	Male	*p* Value
Rectus femoris thickness, cm	1.69 ± 0.35	2.25 ± 0.37	0.001
Vastus intermedius thickness, cm	1.55 ± 0.44	1.94 ± 0.55	0.001
Quadriceps tendon thickness, cm	0.49 ± 0.10	0.60 ± 0.13	0.001
Patellar tendon thickness, cm	0.31 ± 0.05	0.40 ± 0.09	0.001
6 m walk test, s	5.14 ± 0.99	5.10 ± 1.07	0.368
Five-repetition sit-to-stand test, s	12.74 ± 2.67	12.20 ± 2.13	0.318
VAS score	5.08 ± 1.92	4.17 ± 2.47	0.073
WOMAC total score	27.68 ± 16.90	22.84 ± 16.60	0.180
WOMAC pain score	7.11 ± 4.21	5.76 ± 3.55	0.180
WOMAC stiffness score	1.18 ± 1.64	1.55 ± 2.06	0.575
WOMAC function score	19.34 ± 12.33	15.76 ± 11.80	0.185

SD: standard deviation; VAS: Visual Analog Scale; WOMAC: Western Ontario and McMaster Universities Osteoarthritis Index.

**Table 4:** Comparison of ultrasonographic, clinical, and physical performance variables according to BMI category.

Variable	BMI ≤ 25 kg/m^2^	BMI > 25 kg/m^2^	*p* Value
Rectus femoris thickness, cm	1.88 ± 0.44	2.10 ± 0.45	0.023
Vastus intermedius thickness, cm	1.53 ± 0.42	2.01 ± 0.54	0.001
Quadriceps tendon thickness, cm	0.53 ± 0.12	0.57 ± 0.14	0.224
Patellar tendon thickness, cm	0.34 ± 0.06	0.38 ± 0.10	0.028
6 m walk test, s	5.02 ± 0.89	5.24 ± 1.16	0.839
Five-repetition sit-to-stand test, s	12.20 ± 2.19	12.75 ± 2.62	0.434
VAS score	4.56 ± 2.11	4.65 ± 2.45	0.793
WOMAC total score	25.46 ± 17.30	25.03 ± 16.55	0.963
WOMAC pain score	6.66 ± 4.28	6.17 ± 3.51	0.750
WOMAC stiffness score	1.27 ± 1.78	1.49 ± 1.98	0.668
WOMAC function score	17.66 ± 12.27	17.43 ± 12.12	0.971

BMI: body mass index; SD: standard deviation; VAS: Visual Analog Scale; WOMAC: Western Ontario and McMaster Universities Osteoarthritis Index.

**Table 5:** Comparison of ultrasonographic, clinical, and physical performance variables according to smoking status.

Variable	Non-Smoker	Smoker	*p* Value
Rectus femoris thickness, cm	1.90 ± 0.48	2.10 ± 0.41	0.047
Vastus intermedius thickness, cm	1.64 ± 0.48	1.91 ± 0.57	0.041
Quadriceps tendon thickness, cm	0.53 ± 0.11	0.58 ± 0.14	0.148
Patellar tendon thickness, cm	0.35 ± 0.10	0.36 ± 0.06	0.128
6 m walk test, s	5.11 ± 1.15	5.14 ± 0.85	0.668
Five-repetition sit-to-stand test, s	12.34 ± 2.70	12.61 ± 1.95	0.617
VAS score	4.80 ± 2.29	4.32 ± 2.23	0.367
WOMAC total score	26.75 ± 17.89	23.22 ± 15.34	0.484
WOMAC pain score	6.52 ± 4.31	6.31 ± 3.39	0.849
WOMAC stiffness score	1.36 ± 1.88	1.37 ± 1.86	0.875
WOMAC function score	18.82 ± 12.94	15.81 ± 10.85	0.411

Values are presented as mean ± standard deviation. VAS: Visual Analog Scale; WOMAC: Western Ontario and McMaster Universities Osteoarthritis Index.

**Table 6:** Correlations between ultrasonographic measurements and clinical–functional outcomes.

Variable 1	Variable 2	r	*p* Value
Rectus femoris thickness	WOMAC total score	−0.227	0.042
Rectus femoris thickness	WOMAC pain score	−0.233	0.028
Rectus femoris thickness	VAS score	−0.218	0.052
Quadriceps tendon thickness	5× sit-to-stand test	−0.247	0.044
Patellar tendon thickness	Clinical–functional outcomes	Not significant	>0.05

WOMAC: Western Ontario and McMaster Universities Osteoarthritis Index; VAS: Visual Analog Scale.

**Table 7:** Regression analysis of clinical, ultrasonographic, and physical performance variables.

Dependent Variable	Independent Variable	β	*p* Value	R	R^2^
6 m walk test	Five-repetition sit-to-stand test	−0.302	0.007	0.302	0.091
Five-repetition sit-to-stand test	6 m walk test	−0.286	0.008	0.417	0.174
Five-repetition sit-to-stand test	Quadriceps tendon thickness	−0.215	0.044	0.417	0.174
VAS score	Rectus femoris thickness	−0.218	0.052	0.218	0.047
WOMAC pain score	Rectus femoris thickness	−0.252	0.028	0.252	0.064
WOMAC total score	Rectus femoris thickness	−0.258	0.042	0.258	0.066

VAS: Visual Analog Scale; WOMAC: Western Ontario and McMaster Universities Osteoarthritis Index.

## Data Availability

The data supporting the findings of this study are available from the corresponding author upon reasonable request.

## Abbreviations

PFPS	Patellofemoral Pain Syndrome
VAS	Visual Analog Scale
WOMAC	Western Ontario and McMaster Universities Osteoarthritis Index
USG	Ultrasonography
RF	Rectus Femoris
VI	Vastus Intermedius
QT	Quadriceps Tendon
PT	Patellar Tendon
